# PoGO: Prediction of Gene Ontology terms for fungal proteins

**DOI:** 10.1186/1471-2105-11-215

**Published:** 2010-04-29

**Authors:** Jaehee Jung, Gangman Yi, Serenella A Sukno, Michael R Thon

**Affiliations:** 1 Department of Computer Science, Texas A&M University, College Station, TX, 77843, USA; 2 Centro Hispano-Luso de Investigaciones Agrarias (CIALE), Department of Microbiology and Genetics, University of Salamanca, Villamayor, 37185, Spain; 3 Samsung Electronic Co., LTD. 416 Maetan-3dong, Yeongtong-gu, Suwon-City, Gyeonggi-do 443-742, Korea

## Abstract

**Background:**

Automated protein function prediction methods are the only practical approach for assigning functions to genes obtained from model organisms. Many of the previously reported function annotation methods are of limited utility for fungal protein annotation. They are often trained only to one species, are not available for high-volume data processing, or require the use of data derived by experiments such as microarray analysis. To meet the increasing need for high throughput, automated annotation of fungal genomes, we have developed a tool for annotating fungal protein sequences with terms from the Gene Ontology.

**Results:**

We describe a classifier called PoGO (Prediction of Gene Ontology terms) that uses statistical pattern recognition methods to assign Gene Ontology (GO) terms to proteins from filamentous fungi. PoGO is organized as a meta-classifier in which each evidence source (sequence similarity, protein domains, protein structure and biochemical properties) is used to train independent base-level classifiers. The outputs of the base classifiers are used to train a meta-classifier, which provides the final assignment of GO terms. An independent classifier is trained for each GO term, making the system amenable to updating, without having to re-train the whole system. The resulting system is robust. It provides better accuracy and can assign GO terms to a higher percentage of unannotated protein sequences than other methods that we tested.

**Conclusions:**

Our annotation system overcomes many of the shortcomings that we found in other methods. We also provide a web server where users can submit protein sequences to be annotated.

## Background

Our ability to obtain genome sequences is quickly outpacing our ability to annotate genes and identify gene functions. The automated annotation of gene models in newly sequenced genomes is greatly facilitated by easy-to-use software pipelines [1]. While equally as important as gene the gene models, functional annotation of proteins is usually limited to an automatic processing with sequence similarity searching tools such as BLAST, followed by extensive manual curation by dedicated database curators and community annotation jamborees. There is increasing demand to quickly annotate newly sequenced genomes so that they can be used for designing microarray analyses, proteomics, comparative genomics, and other experiments. It is clear that manual gene function annotation cannot be scaled to meet the influx of newly sequenced model organisms.

Two of the main considerations for developing a protein function classifier are the sources of evidence, or features, used for assigning functional categories to the proteins and the method used for defining the relationships between the features and the categories. A number of automated methods have been developed in recent years that vary both in the sources of features used as evidence for the classifier and in the classification algorithm used to assign protein functions. The most common feature types rely on sequence similarity, often using BLAST [2] to identify similar proteins from a large database. Classifiers may also utilize protein family databases such as PFAM and InterPro, bio-chemical attributes such as molecular weight and percentage amino acid content [3], phylogenetic profiles [4,5] transcription factor binding sites [6] as sources of features. Several classifiers have also been developed that utilize features from laboratory experiments such as gene expression profiles [7,8], and protein-protein interactions [8,9].

A classifier is used to determine the relationships between protein features and their potential functions. The simplest examples are manually constructed mapping tables which represent the curator's knowledge of the relationships between features and functional categories. One example is Interpro2GO, a manually curated mapping, of InterPro terms to GO terms. The GOA project relies on several such mappings to provide automated GO annotations for users [10,11]. More sophisticated classifiers have been developed using machine learning algorithms which can automatically deduce the relationships between the features and the functional categories based on a set of previously annotated proteins that serve as training examples. The use of machine learning algorithms can improve the accuracy of the annotations by discovering relationships between features and the functional categories that were not discovered by human curators [12-15]. In addition, they can be applied rapidly and consistently to large datasets, saving many hours of human curation.

Draft genome sequences and machine generated gene models are becoming available for an increasing number of fungal species but machine annotations of protein functions are still limited. We found that pre-existing functional classifiers are either not amenable to genome-scale analyses or are trained only for a small range of taxa [6,16,17]. In addition, many of the previously published protein function annotation systems utilize features obtained from laboratory experiments such as transcriptional profiling or protein-protein interaction assays. These methods cannot be applied to most fungal proteomes since experimental data are not available.

Classifiers that employ features derived from multiple, heterogeneous data sources usually transform the format of each data type into a common format regardless of the properties of each data source [17]. For example, an e-value threshold might be applied to BLAST search results instead of utilizing the e-value directly. The problems with this approach are that the data from each data source is weighted differently and that distinctions between data points can be lost during transformation. Meta-learning classifiers (meta-classifiers) overcome this problem by training independent classifiers (called base-classifiers) for each heterogeneous data source and then use the decisions from the base-classifiers as features to train a meta-classifier. In this way, weights of each data source can be learned by the meta-classifier. Meta-learning classifiers are useful for combining multiple weak learning algorithms and for combining heterogeneous data sources.

We developed a functional classification system called PoGO (Prediction of Gene Ontology terms) that enables us to assign GO terms to fungal proteins in a high-throughput fashion without the requiring evidence from laboratory experiments. We incorporated several evidence sources that emulate what a human curator would use during manual protein function annotation and we avoided features that require laboratory experiments or that otherwise could not be applied to newly sequenced genomes. During the course of our experiments, we discovered that taxon-specific classifiers outperform classifiers that are trained with larger datasets from a wide array of taxa. We developed a taxon-specific classifier for Fungi and we are currently using it to assign GO terms to the proteomes of more than 30 filamentous fungi. We provide as web application that enables users to annotate proteins through their web browser.

## Implementation and Results

### Overview of the PoGO classifier

Protein functional annotation is a multi-label classification problem in which each protein may be assigned one or more GO terms. Various approaches may be applied to solving multi-label classification problems, but the simplest method, and the one we employed, is to consider each GO term as an independent classification problem. Thus, PoGO is actually a team of independent classifiers. The disadvantage to this approach is that interdependencies among the labels cannot be incorporated into the classifier, thus potentially increasing error. The advantage, however, is that a wide range of binary classification algorithms may be applied to the problem. In addition, this approach is more flexible in that different algorithms and/or datasets may be used to train the classifier for each GO term, enabling us to optimize the classification of individual GO terms or groups of GO terms if necessary. Individual GO terms or groups of GO terms may also be re-trained, as new training data of evidence sources become available. PoGO consists of four base classifiers, each of which utilizes distinct data sources, and a meta-classifier for combining the outputs from the base classifiers into a final classification.

### Design and evaluation of the base classifiers

#### InterPro

InterPro terms [18] are defined in the InterPro database which is a curated protein domain database that acts as a central reference for several protein family and functional domain databases, including Prosite, Prints, Pfam, Prodom, SMART, TIGRFams and PIR SuperFamily. We have previously showed that InterPro is an important source of features for identifying GO terms for proteins [19,20]. Using the InterProScan application [21], we can obtain InterPro terms for unannotated proteins. Using InterProScan, we identified 3339 InterPro terms in the fungal protein dataset. We employed Support Vector Machines (SVM) algorithm as the classifier as described previously [19]. For each GO term, we construct a dataset that is comprised of all proteins that are annotated with the GO term (the positive class) and all proteins that are not annotated with the GO term (the negative class). Since the data sets are highly imbalanced, we perform undersampling of the negative class as described previously [19]. This step removes members of the negative class so that the training dataset will have the same number of proteins as the positive class. We also apply Chi-square feature selection to remove features from the classifier that do not contribute to the accuracy of the classifier. Previous results have shown that these steps improve classification accuracy and reduce learning time [19].

#### Sequence Similarity

Several previous methods including GoFigure [14], GOblet [22], and OntoBlast [23] use sequence similarity based on BLAST [2] results as features. GOAnno [24] is also an extension of the similarity-based annotation using hierarchically structured functional categories. In this example, the annotations assigned to the BLAST hits are used as features. We employ a similar approach. The feature set is comprised of hit obtained in a BLAST search of a database of GO annotated proteins (excluding machine annotated terms) using an expect threshold (*E*-value) of 1^-10^. The BLAST database was constructed by extracting proteins from the taxonomic group Fungi from the UniProt database. The resulting feature set contains 3182 features. We employ undersampling and feature selection as described previously, and also utilize the SVM algorithm to train the classifier.

#### Biochemical properties

Other authors have previously shown that biochemical properties of proteins, such as a protein's charge, amino acid composition, are useful for functional classification [25]. The properties that we use in this study include amino acid content, molecular weight, proportion of negatively and positively charged residues, hydrophobicity, isoelectric point and amino acid pair ratio. We use the pepstat and compseq programs in EMBOSS [26] to compute the biochemical properties based on the amino acid sequence of each protein. Unlike the previous datasets, the biochemical properties are not sparse, and are numeric rather than binary. We compared various forms of undersampling and learning methods and found that with this dataset, the Adaboosting method using a linear classifier [27] and using an unbalanced dataset provided the best accuracy (Table S1, Additional File 1).

#### Protein Tertiary Structure

The fourth feature set is protein structure as computed using the HHpred program [28] which is used for protein homology detection and structure prediction using the SCOP database [29]. We use the top 10 selected templates as features and the remaining undefined templates are set to zero for each protein. This dataset is comprised of 8494 binary features. As described previously, we use the SVM algorithm combined with dataset undersampling to create the classifier.

### Design and evaluation of the meta-classifier

**Figure 1:**
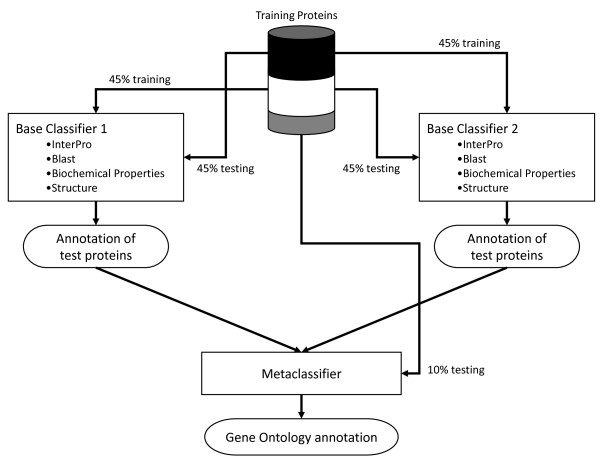
**Overview of the PoGO training and evaluation procedure**. The PoGO classifier uses a Combiner configuration in which two base-level classifiers are trained on 45% of the training proteins and evaluated on another 45% of the training proteins. The remaining 10% of the training proteins are used to evaluate the meta-classifier. This configuration is repeated for each of the Gene Ontology terms trained in PoGO.

The meta-classifier uses the classification decisions from the base classifiers as its inputs (Figure 1). To develop our meta-classifier, we used the Combiner meta-learning strategy [30,31] with a minor modification. Meta-learning strategies such as Combiner are trained using base-level classifiers in which each base-level classifier has been trained using the same input data and different classification algorithms. In our case, we vary both the type of classification algorithm and the type of features used for training, although the set of proteins used to derive that used to train each of the base-classifiers are identical. An overview of the training and classification system is shown in Figure 1. In the case of Combiner, the training dataset *T *is divided into two equal parts *T*_1 _and *T*_2_. The base-level learning algorithms are trained using *T*_1 _and then the resulting classifiers are used to predict functional categories for *T*_2_. Then the process is reversed and *T*_2 _is used for training and *T*_1 _is used for classification. The predictions are then used as features for the meta-classifier. To evaluate the performance of the set of base-classifiers together with the meta-classifier, we perform ten-fold cross validation by constructing ten datasets in which 10% of the proteins are held out of the training dataset. Thus, *T*_1 _and *T*_2 _each contain 45% of the training proteins and the remaining 10% are used for evaluation, in each round of cross validation. We tested two statistical pattern recognition algorithms, Naíve Bayes and Support Vector Machines (SVMs), as meta-classifiers. Since the meta-data are highly imbalanced, we created a balanced dataset by under-sampling as described previously [19] and compared the performance of Naïve Bayes and SVM classifiers trained with both datasets (Table 1). The Naive Bayes classifier trained using the unbalanced dataset provided the best overall performance so it was selected as the meta-classifier.

**Table 1:** Performance comparison of the base and meta classifiers.

Classifier		Sensitivity	Specificity	F-Measure
Base Classifiers	InterPro	0.1893	0.9682	0.2456
	BLAST	0.0251	0.8547	0.0471
	Biochemical properties	0.0099	0.9213	0.0173
	Protein Structure	0.0795	0.9599	0.1304
Meta-classifiers	Naïve Bayes/Unbalanced	0.2620	0.9982	0.3355
	Naïve Bayes/Balanced	0.0629	0.9358	0.1140
	SVM/Unbalanced	0.0011	0.9273	0.0022
	SVM/Balanced	0.0462	0.3696	0.0052

Performance represents an average of all classifiers trained with each data set and is measured by 10-fold cross validation.

### Performance evaluation

Performance evaluation of protein function classifiers performance is a complex issue, since no standard methods exist [32]. Authors typically utilize standard machine-learning metrics such as sensitivity and specificity but the manner in which they are applied vary, which prevents us from directly comparing the performance of classifiers by comparing the performance values reported in publications. Most authors compute performance statistics by first calculating the statistics for each protein individually, and then calculating an average over all of the tested proteins. In a traditional machine-learning approach, performance statistics are calculated for each functional category. The issue is further confounded since the test proteins may be annotated with functional categories that were not within the repertoire of categories that can be predicted by the classifier, and these categories may or may not be treated as classification errors during performance evaluation. Furthermore, the set of proteins used for evaluation is not consistent among all of the protein function classifiers. Authors usually employ cross-validation in order to evaluate their classifier using hold-out sets from the training data set. Standard protein data sets have been developed for annual competitions, such as the one that is often held in conjunction with the Annual International Conference on Intelligent Systems for Molecular Biology (ISMB) but these data sets are not useful for taxon specific classifiers such as PoGO.

Since we train an individual classifier for each functional category, we evaluated the performance of each classifier individually and then computed an arithmetic mean of all the classifiers. All performance metrics were computed using 10-fold cross validation. When we compare the performance of PoGO to other GO term classifiers (AAPFC [19], MultiPfam2GO [33], Gotcha [15], GOPet [13], and InterPro2GO [10]), we use a hold-out set of 71 proteins (1% of our original dataset) that were not used in the training. We calculate the performance of each protein individually, and then report the overall average values. The various classifiers that we tested were all trained using different data sources and were developed to annotate different sets of GO terms. As performance metrics, we use sensitivity, specificity and F-measure. Sensitivity is the proportion of GO term annotations in the training dataset that were correctly annotated by the classifier, and specificity is the proportion of available GO term annotations not assigned to the proteins that were also not assigned to the protein by the classifier. We also use F-measure to provide a single overall metric for comparing the performance of various classifiers.

Considering only the base classifiers, the InterPro term classifier had the highest average F-measure value (Table 1). Surprisingly, the BLAST classifier had low F-measure as well as the lowest specificity, indicating that it results in a large number of false-positive annotations. The F-measure values of the Naïve Bayes meta-classifiers are higher than the base-classifiers (Table 1). The highest F-measure for a meta-classifier (0.3335) was obtained from the Naïve Bayes meta-classifier using the unbalanced dataset. Thus, the meta-classifier trained with the Naïve Bayes algorithm using the unbalanced dataset provides superior results over any single classifier and was used for further experiments.

The classifiers for GO terms that performed poorly were removed by excluding those that have an F-measure below an arbitrary threshold. We tested a range of threshold values and compared the performance to our previous classifier Automated Annotation of Protein Functional Class (AAPFC) [19] which only uses InterPro terms as features. We also compared the performance to MultiPfam2GO [33] a classifier that can assign GO terms to proteins on the basis of protein domains that are described in PFAM. PoGO consistently outperformed AAPFC over the range of threshold values tested (Table 2). The F-measure values obtained with PoGO were comparable to, although slightly lower than, those obtained for MultiPfam2GO. For example, at a threshold value of 0.7, the average F-measure for MultiPfam2GO [33] was 0.99 while the F-Measure for PoGO was 0.98 (Table 2). PoGO was able to assign GO terms to considerably more proteins than either AAPFC or MultiPfam2GO. We attribute this to the additional feature types, such as sequence similarity and protein structure that were included in the dataset. For proteins that to not have matched to the InterPro database, these additional feature types are often times still assigned to the protein, and can be sufficient for predicting the GO terms.

**Table 2:** Overall classifier performance after excluding poorly performing GO term classifiers.

Threshold F-measure	F-Measure			Percentage of Proteins Annotated		
	**AAPFC**	**MultiPfam2GO**	**PoGO**	**AAPFC**	**MultiPfam2GO**	**PoGO**

0.7	0.8955	0.9903	0.9814	1.8	2.8	25.4
0.6	0.8317	0.9705	0.9336	3.8	5.2	31.0
0.5	0.6835	0.9169	0.7518	9.8	10.7	54.3
0.4	0.6619	0.8861	0.6751	17.6	13.4	65.6
0.3	0.5353	0.8520	0.5214	34.9	15.6	86.3
0.2	0.4396	0.5835	0.4456	58.1	28.2	96.2

Properties were computed after removing individual GO term classifiers with an F-measure below the indicated threshold.

We also compared the performance of PoGO to AAPFC, MultiPfam2GO as well as to Gotcha [15], GOPet [13], and InterPro2GO [10] by randomly holding out 1% of the training dataset (71 proteins) prior to training PoGO, and then using the hold-out set to evaluate the performance of all the classifiers. As shown in Table 3, PoGO outperforms the other classifiers by a considerable margin.

**Table 3:** Performance comparison of GO annotation methods using 71 randomly selected proteins.

	Sensitivity	Specificity	F-measure	Percentage of proteins annotated
PoGO	0.3129	0.4101	0.3948	93.0
InterPro2GO	0.0971	0.0938	0.0938	15.5
AAPFC	0.2623	0.3431	0.2217	91.5
Gotcha	0.1182	0.1980	0.1072	100
MultiPfam2GO	0.0945	0.6425	0.1127	64.8
GOPet	0.2080	0.2752	0.1983	100

The evaluation was performed using 71 (1% of the original training dataset) randomly selected proteins that were held out of the training dataset. All GO terms annotated to the evaluation proteins were considered when computing the performance metrics.

### Evaluation of taxon-specific classifiers

Since our goal is to develop a classifier for fungal proteins the previous experiments were performed with a training dataset comprised only of proteins from fungi. Larger training datasets generally result in more robust classifiers so we evaluated the performance of PoGO when non-fungal proteins are included in the training data. We developed a dataset called Fungi-expanded by adding non-fungal proteins to the Fungi dataset that contain at least one GO term that is found in the Fungi dataset. We also prepared another dataset called UniProt that includes all GO term-annotated proteins in the UniProt database regardless of the taxonomic origin. In all cases, we removed GO terms from the proteins that were annotated with the evidence code IEA (Inferred from Electronic Annotation), and those that had less than 10 representative proteins. Among these datasets, Fungi is the smallest. The UniProt set is composed of 119,016 proteins and 2826 GO terms, which is approximately 17 times larger than Fungi in proteins and 7 times larger in GO terms (Table 4). In a similar manner, we prepared three more taxon-specific datasets representing the taxonomic groups Bacteria, Viridiplantae, and Vertebrata.

**Table 4:** Summary statistics and performance evaluation of the taxon-specific classifiers.

Dataset	Proteins	InterPro terms	GO terms	Sensitivity	Specificity	F-measure
UniProt	119016	8414	2826	0.1149	0.9976	0.0893
Fungi	7093	3331	459	0.2680	0.9745	0.3101
Fungi-expanded	70205	5438	1390	0.2304	0.9875	0.2611
Bacteria	3282	2255	115	0.5693	0.9786	0.5691
Viridiplantae	5669	2087	285	0.3808	0.9840	0.3789
Vertebrata	16079	1706	1232	0.0939	0.9555	0.1446

Performance comparison of the InterPro base-classifier on taxon-specific and the UniProt training datasets. No F-measure threshold was applied while computing the performance metrics.

We compared the performance of classifiers trained with each of these data sets using 10-fold cross validation as described previously. Because the training and cross-validation time for all the datasets is prohibitively long we performed these experiments using the InterPro base classifier. We also measured the performance of the Interpro2GO mapping by using it to assign GO terms to proteins and measuring performance with Sensitivity, Specificity, and F-measure. All of the PoGO classifiers outperformed the Interpro2GO mapping (data not shown). Adding additional non-fungal proteins to the fungal specific GO terms (the Fungi-expanded dataset) added more than three times the number of GO terms than the Fungi dataset with only a slight reduction in performance (Table 4). In all cases, the classifiers trained with taxon specific datasets performed better than the classifier trained with the UniProt dataset. Since the Fungi dataset is significantly smaller than the UniProt dataset, we reasoned that the improved performance could be due to overfitting. Overfitting can occur when the training dataset is very small, or the number of features in the model is very large. An overfitted classifier can perform well when the instances (proteins in our case) are similar to the ones in the training data, but perform poorly when presented with instances that are not similar to the training data. To determine whether the improved performance is due to over-fitting, we trained PoGO classifiers with 10 smaller datasets containing the same number of proteins as the Fungi dataset. These subsamples were prepared by randomly selecting proteins from the UniProt dataset. If the classifier for the Fungi dataset was overfitted, we would expect that it would have better performance than the classifiers derived from the sub-sampled datasets. The average F-measure of the subsampled classifiers is 0.4135 while the F-measure for the Fungi classifier is 0.3101, indicating that the Fungi classifier is not subject to overfitting.

An important point to consider is that each GO term is trained independently, and the number of proteins that are used to train each GO term classifier varies considerably, depending on the availability of GO annotated proteins in UniProt. If small data sets lead to overfitting, then we would expect to see a correlation between data set size and classifier performance [34], where classifiers trained with a smaller number of proteins would tend to have higher performance. We compared the performance vs. dataset size for GO terms that were present in the taxon specific and the randomly undersampled datasets but found no correlation in any dataset (Table S2, Additional File 1). The taxon specific datasets may be considered to be a form of data partitioning that removes distantly related proteins from the data. This, in effect, would cause GO terms that are never found within a taxonomic group to be removed from the classifier, thus the error of the individual GO term classifier does not contribute to the overall error of the system.

### Web Server

**Figure 2:**
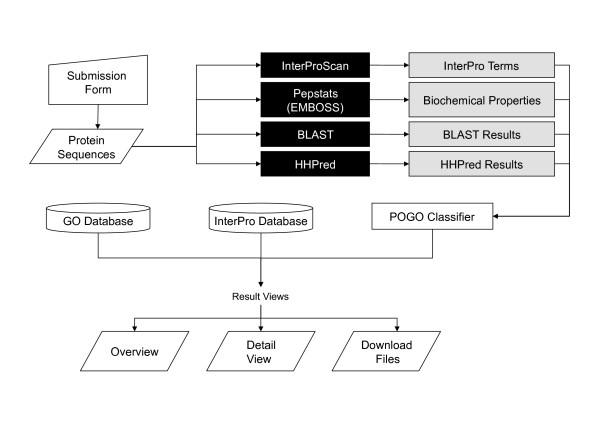
**Flowchart of the PoGO web server**. Four different sequence analysis programs converts data to InterPro term, Blast result, Bio-chemical property and protein structure information, which is represented by the gray and black box. After the transformed data applied to the PoGO training model, we can get the final GO annotation and its supplementary information in each query protein.

We have developed a web server that enables users to annotate protein sequences using the taxon specific classifiers described in this manuscript (Figure 2). The classification results are stored on the server in flat files, and are presented to the user in a table that contains the annotated GO terms as well as and InterPro terms and links to a detailed page for each protein. The detail page contains a graphical view of the InterPro annotations and along with a list of predicted GO terms and descriptions including the category. In addition, the tab-separated data files of the GO annotations are available for download, enabling to import of the data into other programs. The classification algorithms, undersampling, feature selection and the computation of performance metrics were performed in MATLAB [35] using the pattern recognition toolbox [36]. The web application, as well as several data handling scripts, were written in php. The source code for the web server have been released under the terms of the BSD license and is available in Additional File 2 as well as on the PoGO home page.

## Conclusions

In this paper, we describe a meta-classifier for assigning GO terms to proteins using heterogeneous feature sets. By using multiple, heterogeneous data sources, we developed a classifier that offers improved accuracy compared to previously reported annotation methods. More importantly, this system can assign GO terms to a higher proportion of proteins than annotation methods that rely only on one feature type. We also found that taxon-specific classifiers have significantly better performance than species independent systems. Functional annotations provided by PoGO are being used for fungal comparative genomics projects in our research group. We also provide a public web server where users can annotate protein sequences.

## Availability and requirements

Project Name: PoGO: Prediction of gene ontology terms

Project Home page: http://bioinformatica.vil.usal.es/lab_resources/pogo/

Operating system: OS independent. Use any modern web browser

Programming language: MATLAB, php

Other requirements: None

License: BSD

Restrictions for non-academic use: None

## Abbreviations

SVM: Support Vector Machines; GO: Gene Ontology.

## Authors' contributions

JJ proposed the learning scheme and implemented the classifier; GY contributed to the implementation of the web site; MT suggested the basic idea and guided the overall system. JJ, GY, SS and MT wrote and revised the manuscript. All authors have read and approved the final manuscript.

## Supplementary Material

Additional file 1 **Supplementary Tables**.Table 1 - Comparison of classifier methods for assigning Gene Ontology (GO) terms using biochemical properties. Table 2 - Performance comparison of four different taxon-specific data sets and randomly undersampled UniProt data sets. Click here for file

Additional file 2 Software source code and data files. Click here for file
