# Supplementary material for: PoGO: Prediction of Gene Ontology terms for fungal proteins
Source: BMC Bioinformatics. 2010 Apr 29;11:215. doi: 10.1186/1471-2105-11-215 (PMC2882390; doi:10.1186/1471-2105-11-215)
Supplement: Additional file 1 — Supplementary Tables.Table 1 - Comparison of classifier methods for assigning Gene Ontology (GO) terms using biochemical properties. Table 2 - Performance comparison of four different taxon-specific data sets and randomly undersampled UniProt data sets. [file 1471-2105-11-215-S1.PDF]

Supplementary Table 1 - Comparison of classifier methods for assigning Gene Ontology (GO) terms using biochemical properties. Support Vector Machines was compared to Adaboosting using balanced and unbalanced datasets. The unbalanced datasets were constructed by including all proteins annotated with a given GO term as well as 7 fold more proteins not annotated with the given GO term. The balanced datasets were comprised of equal numbers of proteins annotated and not annotated with the given GO term. Adaboosting using the unbalanced data set provided the best performance and was used in PoGO. Values in the table represent mean values for all GO term classifiers tested.

	Sensitivity	Specificity	F-measure
SVM	0.0060	0.6302	0.0118
Adaboosting Balanced	0.0054	0.5123	0.0106
Adaboosting Unbalanced	0.0099	0.9213	0.0173

Supplementary Table 2 - Performance comparison of four different taxon-specific data sets and randomly undersampled UniProt data sets.

This table shows the performance and number of proteins for selected GO terms which are shared by all four taxon-specific and a randomly selected sub-sample of the UniProt data set.

	Bacteria		Plant		Vertebrata		Fungi		subsampled Uniprot	
	Positive	F-measure	Positive	F-measure	Positive	F-measure	Positive	F-measure	Positive	F-measure
GO:0003677	24	0.2423	430	0.6798	538	0.1905	64	0.2137	263	0.7737
GO:0003723	11	0.7841	46	0.5861	286	0.2176	77	0.3268	90	0.5184
GO:0003735	313	0.9421	264	0.9192	121	0.1619	146	0.6521	18	0.1170
GO:0003743	16	0.3327	30	0.2720	41	0.1268	26	0.1150	70	0.4681
GO:0003899	27	0.8301	16	0.8069	20	0.1402	35	0.3111	133	0.9835
GO:0005524	49	0.4402	73	0.3273	430	0.3192	24	0.1139	1074	0.9526
GO:0005525	22	0.5336	43	0.7275	124	0.3458	10	0.1349	177	0.8228
GO:0005576	11	0.6323	19	0.1760	670	0.2356	33	0.2639	59	0.4639
GO:0005737	145	0.7591	162	0.2115	1564	0.2933	925	0.6129	123	0.4383
GO:0006118	20	0.4037	88	0.1949	101	0.1546	11	0.2952	115	0.8340
GO:0006281	48	0.6888	20	0.3939	88	0.1526	60	0.2611	156	0.5330
GO:0006350	29	0.7634	12	0.3788	25	0.0741	20	0.1284	68	0.702
GO:0006355	36	0.4696	312	0.5308	416	0.2543	22	0.0762	69	0.3813
GO:0006412	283	0.8669	257	0.8902	155	0.1967	177	0.7963	1004	0.8593
GO:0006413	16	0.3496	20	0.2952	27	0.2166	32	0.1356	67	0.3871
GO:0006414	23	0.4656	18	0.6345	11	0.0368	10	0.0455	76	0.6077
GO:0006457	38	0.6817	39	0.6495	92	0.2128	50	0.2541	105	0.6019
GO:0006508	36	0.3186	112	0.3266	250	0.3178	23	0.1489	56	0.6443
GO:0006950	29	0.6980	29	0.2825	117	0.1064	68	0.2820	12	0.1579
