# Supplementary material for: PoGO: Prediction of Gene Ontology terms for fungal proteins
Source: BMC Bioinformatics. 2010 Apr 29;11:215. doi: 10.1186/1471-2105-11-215 (PMC2882390; doi:10.1186/1471-2105-11-215)
Supplement: Additional file 2 — Software source code and data files. [file 1471-2105-11-215-S2.GZ › PoGO-1.0.2/website/chem_info.php]

Chemical Information


php
function chem\_show($path\_name){
$chemfile = @fopen($path\_name."/chemratio.out", "r");
if (!$chemfile) {
echo "Unable to open a result file.<br  
\n";
exit;
}
while (!feof($chemfile)) {
$line = fgets($chemfile, 1024\*15);
if( trim($line) !="" ){
$tmp = split(",",$line);
}
}
$chemout= @fopen($path\_name."/chem.out", "r");
if (!$chemout) {
echo "Unable to open a result file.  
  
\n";
exit;
}
while (!feof($chemout)) {
$line = fgets($chemout, 1024\*15);
if( trim($line) !="" ){
$tmpout = split(",",$line);
}
}
$charletter=array("A","C","D","E","F","G","H","I","K","L","M","N","P","Q","R","S","T","U","V","W","Y");
for ($i=0; $iResidue Molecular Percent and Property Molecular Percent

|  |  |  |  |
| --- | --- | --- | --- |
|");
for ($k=0 ; $k< count($charletter)/7 ; $k++){
for ($i= $k\* (7); $i<$k \*(7)+7; $i++){
echo(" ". $charletter[$i]." |");
}
echo ("|");
for ($i= 2+$k\*7; $i<2+$k\*7+7; $i++){
echo(" ". $tmpout[$i]." |");
}
echo ("|");
}
for ($k=0 ; $k< count($petstat)/7 ; $k++){
for ($i= $k\* (7); $i<$k \*(7)+7; $i++){
echo(" ". $petstat[$i]." |");
}
echo ("|");
for ($i= 24+$k\*7; $i<24+$k\*7+7; $i++){
echo(" ". $tmpout[$i]." |");
}
echo ("|");
}
echo("

  
  
 Chemical Ratio

|  |  |
| --- | --- |
|");
for ($k=0 ; $k< count($charlist)/7 ; $k++){
for ($i= $k\* (7); $i<$k \*(7)+7; $i++){
echo(" ". $charlist[$i]." |");
}
echo ("|");
for ($i= $k\*7; $i<$k\*7+7; $i++){
echo(" ". $tmp[$i]." |");
}
echo ("|");
}
echo("

");
}
$path\_id = $\_REQUEST["pathid"];
if( trim($path\_id) != "" ) {
chem\_show($path\_id);
} else {
echo "GO term is required to show its information.";
}
?>
