# Supplementary figures and images for: PoGO: Prediction of Gene Ontology terms for fungal proteins
Source: BMC Bioinformatics. 2010 Apr 29;11:215. doi: 10.1186/1471-2105-11-215 (PMC2882390; doi:10.1186/1471-2105-11-215)

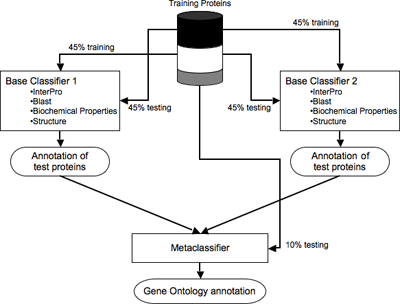

Supplement: Additional file 2 — Software source code and data files. [file 1471-2105-11-215-S2.GZ › PoGO-1.0.2/website/menu/fig1.png]

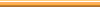

Supplement: Additional file 2 — Software source code and data files. [file 1471-2105-11-215-S2.GZ › PoGO-1.0.2/website/img/bar1.gif]

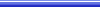

Supplement: Additional file 2 — Software source code and data files. [file 1471-2105-11-215-S2.GZ › PoGO-1.0.2/website/img/bar10.gif]

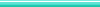

Supplement: Additional file 2 — Software source code and data files. [file 1471-2105-11-215-S2.GZ › PoGO-1.0.2/website/img/bar11.gif]

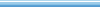

Supplement: Additional file 2 — Software source code and data files. [file 1471-2105-11-215-S2.GZ › PoGO-1.0.2/website/img/bar12.gif]

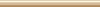

Supplement: Additional file 2 — Software source code and data files. [file 1471-2105-11-215-S2.GZ › PoGO-1.0.2/website/img/bar13.gif]

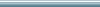

Supplement: Additional file 2 — Software source code and data files. [file 1471-2105-11-215-S2.GZ › PoGO-1.0.2/website/img/bar2.gif]

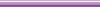

Supplement: Additional file 2 — Software source code and data files. [file 1471-2105-11-215-S2.GZ › PoGO-1.0.2/website/img/bar3.gif]

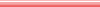

Supplement: Additional file 2 — Software source code and data files. [file 1471-2105-11-215-S2.GZ › PoGO-1.0.2/website/img/bar4.gif]

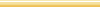

Supplement: Additional file 2 — Software source code and data files. [file 1471-2105-11-215-S2.GZ › PoGO-1.0.2/website/img/bar5.gif]

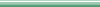

Supplement: Additional file 2 — Software source code and data files. [file 1471-2105-11-215-S2.GZ › PoGO-1.0.2/website/img/bar6.gif]

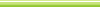

Supplement: Additional file 2 — Software source code and data files. [file 1471-2105-11-215-S2.GZ › PoGO-1.0.2/website/img/bar7.gif]

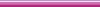

Supplement: Additional file 2 — Software source code and data files. [file 1471-2105-11-215-S2.GZ › PoGO-1.0.2/website/img/bar8.gif]

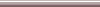

Supplement: Additional file 2 — Software source code and data files. [file 1471-2105-11-215-S2.GZ › PoGO-1.0.2/website/img/bar9.gif]

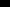

Supplement: Additional file 2 — Software source code and data files. [file 1471-2105-11-215-S2.GZ › PoGO-1.0.2/website/img/ending.gif]

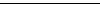

Supplement: Additional file 2 — Software source code and data files. [file 1471-2105-11-215-S2.GZ › PoGO-1.0.2/website/img/mid.gif]

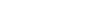

Supplement: Additional file 2 — Software source code and data files. [file 1471-2105-11-215-S2.GZ › PoGO-1.0.2/website/img/whitebar.gif]

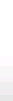

Supplement: Additional file 2 — Software source code and data files. [file 1471-2105-11-215-S2.GZ › PoGO-1.0.2/website/images/bottom_table_bg.gif]

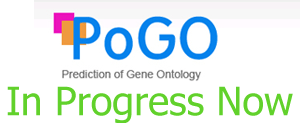

Supplement: Additional file 2 — Software source code and data files. [file 1471-2105-11-215-S2.GZ › PoGO-1.0.2/website/images/InProgressNowLogo.gif]

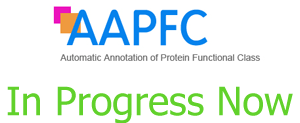

Supplement: Additional file 2 — Software source code and data files. [file 1471-2105-11-215-S2.GZ › PoGO-1.0.2/website/images/InProgressNowLogo_AAPFC.gif]

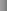

Supplement: Additional file 2 — Software source code and data files. [file 1471-2105-11-215-S2.GZ › PoGO-1.0.2/website/images/L_V.gif]

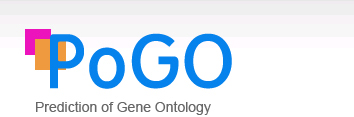

Supplement: Additional file 2 — Software source code and data files. [file 1471-2105-11-215-S2.GZ › PoGO-1.0.2/website/images/Logo.jpg]

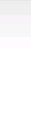

Supplement: Additional file 2 — Software source code and data files. [file 1471-2105-11-215-S2.GZ › PoGO-1.0.2/website/images/Logo_bg.jpg]

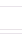

Supplement: Additional file 2 — Software source code and data files. [file 1471-2105-11-215-S2.GZ › PoGO-1.0.2/website/images/menu_bg.gif]

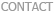

Supplement: Additional file 2 — Software source code and data files. [file 1471-2105-11-215-S2.GZ › PoGO-1.0.2/website/images/menu_contact.gif]

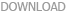

Supplement: Additional file 2 — Software source code and data files. [file 1471-2105-11-215-S2.GZ › PoGO-1.0.2/website/images/menu_download.gif]

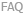

Supplement: Additional file 2 — Software source code and data files. [file 1471-2105-11-215-S2.GZ › PoGO-1.0.2/website/images/menu_faq.gif]

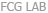

Supplement: Additional file 2 — Software source code and data files. [file 1471-2105-11-215-S2.GZ › PoGO-1.0.2/website/images/menu_fcg_lab.gif]

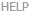

Supplement: Additional file 2 — Software source code and data files. [file 1471-2105-11-215-S2.GZ › PoGO-1.0.2/website/images/menu_help.gif]

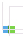

Supplement: Additional file 2 — Software source code and data files. [file 1471-2105-11-215-S2.GZ › PoGO-1.0.2/website/images/menu_left.gif]

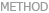

Supplement: Additional file 2 — Software source code and data files. [file 1471-2105-11-215-S2.GZ › PoGO-1.0.2/website/images/menu_method.gif]

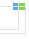

Supplement: Additional file 2 — Software source code and data files. [file 1471-2105-11-215-S2.GZ › PoGO-1.0.2/website/images/menu_right.gif]

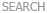

Supplement: Additional file 2 — Software source code and data files. [file 1471-2105-11-215-S2.GZ › PoGO-1.0.2/website/images/menu_search.gif]

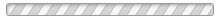

Supplement: Additional file 2 — Software source code and data files. [file 1471-2105-11-215-S2.GZ › PoGO-1.0.2/website/images/progress_bar.gif]

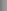

Supplement: Additional file 2 — Software source code and data files. [file 1471-2105-11-215-S2.GZ › PoGO-1.0.2/website/images/R_V.gif]

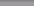

Supplement: Additional file 2 — Software source code and data files. [file 1471-2105-11-215-S2.GZ › PoGO-1.0.2/website/images/table_bottom_bar_bg.gif]

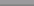

Supplement: Additional file 2 — Software source code and data files. [file 1471-2105-11-215-S2.GZ › PoGO-1.0.2/website/images/table_top_bar_bg.gif]

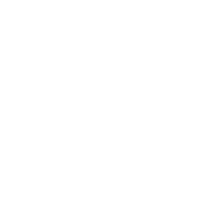

Supplement: Additional file 2 — Software source code and data files. [file 1471-2105-11-215-S2.GZ › PoGO-1.0.2/website/images/warnning.gif]

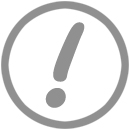

Supplement: Additional file 2 — Software source code and data files. [file 1471-2105-11-215-S2.GZ › PoGO-1.0.2/website/images/warnning.jpg]
